# A computational study of light-induced superimposed mechanical and dipolar effects

**DOI:** 10.1140/epjp/s13360-025-06866-0

**Published:** 2025-09-30

**Authors:** Fabio Marangi, Giulia Simoncini, Chiara Florindi, Francesco Lodola, Giuseppe Maria Paternò, Guglielmo Lanzani

**Affiliations:** 1 Center for Nano Science and Technology, Istituto Italiano di Tecnologia (IIT), Via Rubattino, 81, 20134 Milan, Italy; 2 Department of Physics, Politecnico di Milano, Piazza L. da Vinci 32, 20133 Milan, Italy; 3 Department of Biotechnology and Biosciences, University of Milan-Bicocca, Building U3 - BIOS, Piazza della Scienza, 2, 20126 Milan, Italy

## Abstract

Light-sensitive molecules provide a powerful means to control cellular excitability without genetic modification. Among them, the amphiphilic membrane targeting azobenzene Ziapin2 has emerged as a versatile photo-switch able to modulate membrane potential. Previous studies have attributed its action mainly to an opto-mechanical effect. However, azobenzenes are known to undergo significant light-induced dipole changes, raising the possibility of additional electrical contributions. Here, we combine experimental data and numerical modeling to investigate this dual mechanism in Ziapin2. Our analysis shows that beyond capacitance modulation, a substantial increase in molecular dipole moment (> 6D) can shift membrane surface potential, partially counteracting the hyperpolarizing effect. A model with time-varying surface potential captures key features of published responses and shows that polarity is governed by the membrane interface at which the photo-dipole is expressed, not by the dipole change alone. This combined framework provides a more complete description of Ziapin2 action and enables prospective design of next-generation molecules with tailored selective depolarizing or hyperpolarizing response.

## Introduction

Light-sensitive molecules have emerged as powerful exogenous non-genetic tools for the remote control of cellular excitability [1–3]. Among them, the azobenzene molecule Ziapin2 has been shown to partition into lipid bilayers, in which it maintains its photo-isomerization capability [4, 5]. This *trans → cis* photo-switching drives a rapid mechanical perturbation of the lipid membrane [6], producing a change in membrane capacitance and a subsequent hyperpolarizing voltage transient that is sufficient to modulate neuronal firing [7] and muscular contraction [8, 9]. Numerical models of Ziapin2 photo-stimulation mechanism have so far been built on this opto-mechanical capacitance effect alone [10, 11], treating the molecule as a reversible actuator of bilayer thickness [12].

However, photo-isomerization of azobenzene derivatives is also known to alter their molecular dipole moments [2]. In another membrane-targeted photo-switch, MTP2, light-induced dipole change has been utilized to elicit cellular response. In fact, changes in surface potential generated by a large decrease in dipole moment led to a membrane depolarization, without soliciting other mechanical effects [13].

**Fig. 1:**
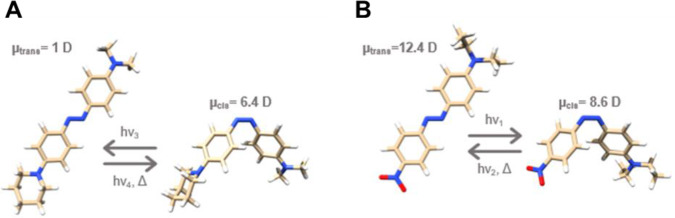
Molecular structures and dipoles. DFT calculation of the *trans*- to *cis*-variation of the dipole moment of representative molecular models of Ziapin2 (**A**) and MTP2 (**B**). Adapted from Sesti et al. [13]

Although Ziapin2 literature has generally assumed that dimerization cancels out dipolar contributions, quantum-chemical calculations reveal a > 6D increase in dipole moment [13] upon isomerization (Fig. 1A), indicating that a dipole-driven surface potential change should, in principle, accompany the capacitance effect. In this framework, via numerical modeling, we want to explore the possibility that Ziapin2’s opto-mechanical action is systematically weakened by a hidden dipolar pathway: While the capacitance change tends to hyperpolarize, the simultaneous increase in molecular dipole moment may partially counteract this by shifting the membrane surface potential in the opposite direction. We believe that the disentanglement of these two mechanisms is crucial for understanding the experimental response of cells to Ziapin2 and for guiding the design of next-generation tools with tailored electrical effects.

## Simulation results and discussion

A simple equivalent circuit model has been proposed in the literature to describe membrane parameters of cells [14]. Previous work with Ziapin2 [10, 11] on HEK-293 cells showed that the electrophysiological response upon photo-isomerization could be reproduced by incorporating a step-like function into the model to simulate light-induced changes in membrane capacitance (*C*_m_). The rising and falling time constants were closely related to the concentration of the *cis-*isomer, as determined by optical measurements. Similarly, the MTP2 response was simulated by keeping the membrane capacitance fixed and introducing an additional generator in the circuit (surface potential, *V*_s_), whose value varied in a step-like manner upon light excitation. However, a simultaneous combination of effects requires the introduction of two time-dependent terms, namely *C*_m_(*t*) and *V*_s_(*t*), and the solution of the ordinary differential equation:

1 $$\frac{{{\text{d}}V_{{\text{m}}} }}{{{\text{d}}t}} = \frac{{\partial V_{{\text{S}}} }}{\partial t} - \frac{{\partial {\text{C}}_{{\text{m}}} }}{\partial t} \cdot \frac{{V_{{\text{m}}} - V_{{\text{S}}} \left( t \right)}}{{C_{{\text{m}}} \left( t \right)}} + \frac{{V_{{\text{r}}} - V_{{\text{m}}} }}{{C_{{\text{m}}} \left( t \right)R_{{\text{m}}} }}$$

In this model *V*_r_ and *R*_m_ represent the resting membrane potential and membrane resistance, respectively. Here, *R*_m_ is treated as a constant, which is a reasonable assumption for simple HEK-293T cells, as it avoids the need to account for voltage-gated ion channels activation and the resulting ionic currents.

**Fig. 2:**
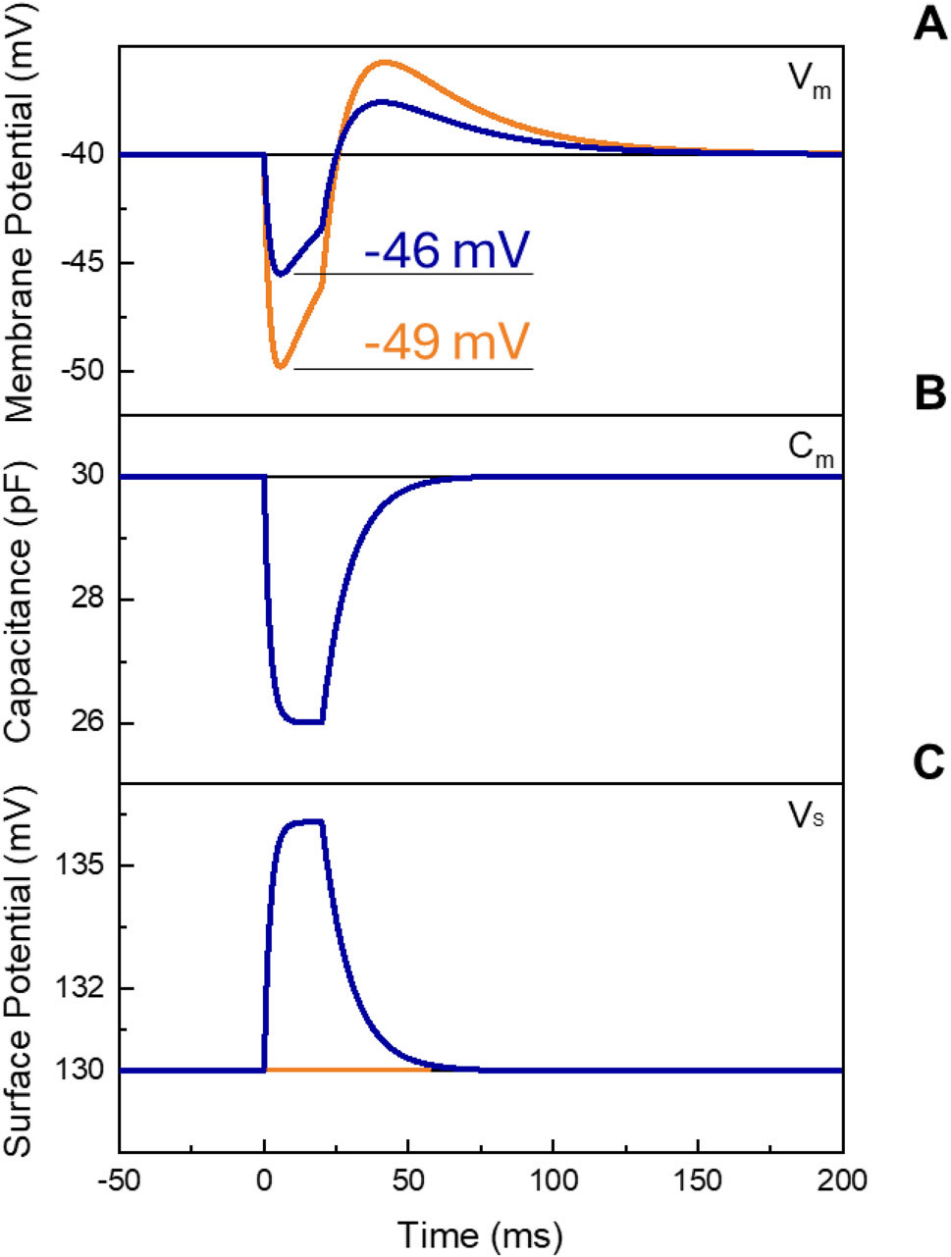
Membrane potential variation. (**A**) *V*_m_ variation over time upon 20 ms light stimulation with (blue) and without (orange) considering the variation of *V*_s_. (**B**) Capacitance and (**C**) surface potential variation over time

Figure 2 reports the simulation results for HEK-293 cells treated with Ziapin2 (25 μM) and excited with a blue (470 nm) light pulse of 20 ms at a power density of 50 mW/mm^2^. The variation of the parameters shows how the simultaneous variation of both *C*_m_ and *V*_s_ occurs with similar time constants. By considering a − 15% (4 pF) variation of *C*_m_ and a + 5% variation of *V*_s_ (5 mV), the shape and intensity of the response are consistent with those previously observed experimentally and reported in numerical simulations which did not consider the coexistence of the two phenomena. Moreover, the current numerical model more closely reproduces the depolarization rebound, which not only depends on the cellular bio-electric machinery, but also on the reorganization of surface charges. Even if not previously considered, Ziapin2 photo-isomerization process leads to a sixfold increase of molecular dipole moment. Although with different sign, a smaller difference in dipole intensity has been demonstrated to be sufficient for the MTP2-induced depolarization of membrane potential in experiments on HEK-293 cells, in which with the same conditions a—4D difference between *trans*- and *cis*-isomers (Fig. 1B) led to a 5 mV increase in *V*_s_. Hence, in the case of Ziapin2, the change in molecular dipole moment of more than 6D corresponds to a membrane potential shift of Δ*V*_m_ = − 3 mV when the dipolar effect is considered compared to when it is not.

The discrepancy in the dipole sign change / depolarization effect between Ziapin2 and MTP2 can be explained by the increased affinity of the first to the outer leaflet. In fact, comparing the molecular structure of Ziapin2 and MTP2, the presence of a strong electron-withdrawing group as NO_2_ in the latter results in a much stronger molecular dipole of the *trans-*MTP2 (12.4D) [13] with respect to the *trans-*Ziapin2 (1.05D) [13]. Consequently, MTP2 flip-flop events will be more likely to occur, while by contrast Ziapin2’s weak *trans-* dipole may result in minimal equilibrium dipole alignment forces, thus favoring chemical anchoring of lipid polar heads and the grafting groups of the molecule.

**Fig. 3:**
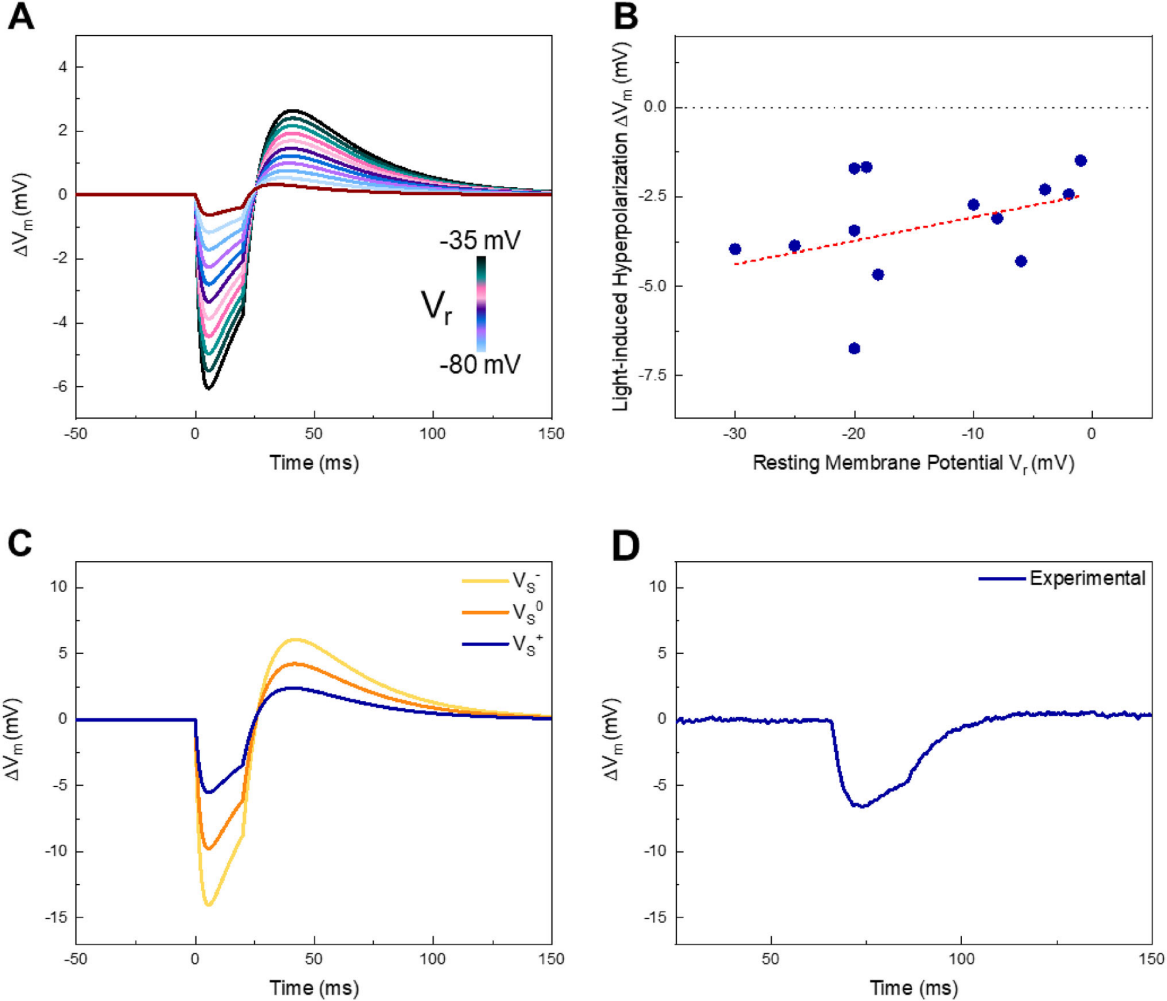
Effect of resting potential and *V*_s_ polarity. (**A**) Membrane potential traces for holding potentials from − 35 to − 85 mV in the two-pathway model. (**B**) Amplitude of light-induced hyperpolarization as a function of *V*_r_, in HEK-293T loaded with Ziapin2 (25 μM) and stimulated with 470 nm light pulses of 20 ms at 50 mW/mm^2^. The dashed line represents the linear regression curve of the plotted data points (*R*^2^ = 0.45). (**C**) Membrane potential traces considering a positive (blue), negative (yellow) and null (orange) variation of *V*_s_. (**D**) Representative experimental trace of HEK-293T (*V*_r_ = − 21 mV) loaded with Ziapin2 (25 μM) and stimulated with 470 nm light pulses of 20 ms at 50 mW/mm^2^. All traces have been zeroed for representation purposes

As a result, flip-flop events can happen on longer timescales and lead to molecular dimerization as previously reported. However, an excess of molecules may be found preferentially in the outer leaflet and can contribute to the membrane potential response upon photo-stimulation opposing to the opto-mechanical effect. When we varied *V*_r_ in the simulations, the magnitude of the light-evoked deflection increased as the cell was held more negative (Fig. 3A)—simulating a stronger inward transmembrane field, hence a more effective molecular dipoles alignment. Simulation results find confirmation in experimental data. The correlation between cell *V*_r_ and light-induced hyperpolarization amplitude reported in Fig. 3B displays the same trend predicted by the simulations, thus validating the assumptions on molecular dipoles alignment and leaflet occupancy.

Figure 3C reports numerical traces isolating the contribution of different design parameters. The orange curve corresponds to Ziapin2 with the opto-mechanical pathway only (no dipole contribution). The blue trace utilizes the same parameters but with the addition of a depolarizing dipole-induced surface potential, representing the real conditions. In this case, the superimposed dipolar effect cancels a large fraction of the early membrane stretching-induced hyperpolarization. Conversely, the yellow trace has been obtained by imposing a hyperpolarizing dipolar term, showing that the two effects can be combined to generate stronger stimuli. The representative trace in Fig. 3D has been extracted from an HEK-293T (*V*_r_ = − 21 mV) loaded with Ziapin2 (25 μM) and stimulated with 470 nm-light pulses of 20 ms at 50 mW/mm^2^. As predicted, the shape of the curve and light-induced hyperpolarization value of ~ − 6 mV are very close to the ones predicted by the mathematical model.

## Conclusion

In this literature-based analysis, we have uncovered an underlying phenomenon that influences the cellular response. We assumed that leaflet fractions are quasi-static on the timescale of the pulse (consistent with slow flip-flop events) and that the coupling between dipole change and surface potential can be well-captured by a single amplitude parameter scaling with field strength. These assumptions might be relaxed in future work by introducing explicit orientation and leaflet kinetics, potentially revealing a preference for accumulation in the outer leaflet. Such refinement would help explain the observed increase in response magnitude at more negative holding potentials, a hallmark also reported for other membrane-targeted azobenzene derivatives whose action is primarily dipole-driven.

Importantly, the polarity of the surface potential step is not determined by the sign of the dipole change alone. Specifically, it depends on how the effective dipole projects onto the membrane normal and on which leaflet is enriched. For Ziapin2, preferential occupancy of the outer leaflet with outward-pointing dipoles produces a depolarizing surface potential step upon light irradiation. Accordingly, the preferred inner leaflet positioning of MTP2 [13], combined with inward pointing dipoles and a light-induced molecular dipole shortening, also yields a depolarizing step. In both cases, stronger fields amplify voltage changes by enhancing dipole alignment. Consequently, for Ziapin2, the dipolar depolarization partially cancels the fast mechanical hyperpolarization, reducing its impact and giving rise to a depolarizing plateau, which not only depends on cellular adaptation mechanisms, but also emerges from the interplay between dipolar and mechanical contributions.

Most importantly, by accounting for the dipolar contribution and its dependence on leaflet occupancy and orientation, we move from post hoc interpretation to prospective design. Substituents can be selected to obtain specific Δµ, while anchoring and partitioning strategies can localize the effect to either the outer or inner leaflet, thereby programming depolarizing or hyperpolarizing photo-responses on demand. This insight enables the rational design of new photo-switches to elicit targeted electrical effects, whether stronger depolarization, stronger hyperpolarization, or tuned cancellation, through systematic control of substituents (Δμ), leaflet bias, and orienting groups.

## Methods

### HEK-293T culture

Electrophysiological experiments were conducted in vitro using the immortalized Human Embryonic Kidney cell line HEK-293T (purchased from ATCC). Cells were maintained in T-25 culture flasks containing Dulbecco’s modified eagle medium, high-glucose (DMEM-HG) culture medium, supplemented with 10% heat-inactivated fetal bovine serum (FBS) and 1% GlutaMAX (200 mM). Cultures were incubated at 37 °C in a humidified atmosphere with 5% CO_2_. Once cells reached 80% confluence, they were detached using 1× trypsin–EDTA solution, seeded onto sterilized substrates, and allowed to grow for 24 h. To enhance cell adhesion, substrates were pre-coated with a layer of fibronectin (2 μg/mL in PBS buffer solution) and incubated for 1 h at 37 °C before plating. All reagents were purchased from Invitrogen.

### Ziapin2 uptake

HEK-293T cells, pre-seeded in Petri dishes, were incubated with 25 µM Ziapin2 for 7 min at room temperature, avoiding direct light exposure. After the incubation, media was replaced with fresh extracellular solution to remove any uninternalized molecules.

### Patch-clamp electrophysiology

Whole-cell current-clamp recordings were performed using freshly pulled glass pipettes (4–7 MΩ) filled with an intracellular solution containing (mM): 125 K-gluconate, 12 KCl, 1 MgCl_2_, 0.1 CaCl_2_, 10 EGTA, 10 HEPES, and 10 Na_2_-ATP (pH 7.3 with KOH). Cells were continuously superfused at 36 °C with an extracellular solution containing (mM): 135 NaCl, 5.4 KCl, 1.8 CaCl_2_, 1 MgCl₂, 10 glucose, and 5 HEPES (pH 7.4 with NaOH). Signals were acquired with a MultiClamp 700 A amplifier (Molecular Devices), digitized at 20 kHz using an Axon Digidata 1440A, and filtered at 10 kHz. During electrophysiological recordings, cells were illuminated using a collimated light-emitting diode (LED, Thorlabs) coupled to the fluorescence port of a Nikon Eclipse TE200 inverted microscope. The LED provided excitation at 470 nm, matching the absorption spectrum of the molecule. Illumination was delivered over a 0.27 mm^2^ spot with a power density of 50 mW/mm^2^, quantified at the microscope objective using an optometer.

## Data Availability

Data sets generated during the current study and experimental data are available from the corresponding author on reasonable request. The manuscript has associated data in a data repository.
